# The Alumni Reunion

**Published:** 1900-03

**Authors:** 


### THE ALUMNI REUNION.

THE annual reception by the faculty to the alumni of the Medical Department of the University of Buffalo, a custom inaugurated last year, was held in the Alumni Hall of the University building, Thursday evening, February 22, 1900.

A large audience, generously besprinkled with ladies, graced the occasion, and the hall was appropriately decorated with patriotic emblems, reminding all that Washington's birthday had been appropriated by the faculty as permanent University day.

The dean of the faculty, Dr. M. D. Mann, was greeted with a round of applause when he took the chair and introduced the speaker of the evening, Dr. Frederick Peterson, '79, of New York, whose subject was Practical psycology. To say that it was interestingly instructive, even ably dealt with only echoes the universal opinion of everyone who heard the distinguished visitor. Dr. Peterson was invited to address the meeting last year, but was prevented by illness. He was, therefore, doubly greeted when he appeared with a robustness that was indicative of health, happiness, and longevity. His address will be published in an early issue of the JOURNAL.

The next feature of the evening was an attempt by Madame Bergman, of Buffalo, to demonstrate what is termed psychometry—whatever that may mean. At all events the famous "mind-reader" made sorry work of her attempt to measure the mentalities of several of her prominent auditors. It was easy to see how, by her jugglery with words and by making a few common-place guesses, more or less accurate, the superficially trained—even the superrefined—might be impressed with the wonderful "gifts" of such a person. It was a "demonstration" that in this instance failed to demonstrate.

Dr. Jason Parker, of Jamestown, who was billed to give a practical demonstration of hypnotism, was more successful. From a group of messenger boys hastily collected, he succeeded in apparently hypnotising a number of them. As an exhibition it was entertaining and succeeded in stimulating speculative thoughts in the minds of many present as to its applicability or utility. Dr. Parker received the applause of his audience many times during his entertaining demonstration.

Then came Prof. Gee Hart, of Buffalo, who delighted everybody by a sleight-of-hand performance. Gee-whiz! how he did fill the air with half-dollars, all of which rained into his own coffers. Some one suggested that he ought to get a permanent place as a passer of the plate in our most fashionable churches. Frequent applause testified to the interest in this part of the entertainment.

This closed the exercises of the evening in Alumni Hall, after which luncheon was served in the library where a pleasant hour was passed. The executive committee deserves credit for providing a unique and entertaining program, which was appreciated by the audience.

It is to be regretted that Governor Roosevelt, who was on a visit to Buffalo, could not attend. He signified his willingness to do so and say a few words, provided the Dean of the Saturn Club and the Colonel of the 65th Regiment, in whose hands he was for the evening would consent. But the JOURNAL is informed that neither Mr. Douglass nor Col. Welch—one or both— could be prevailed upon to relinquish their claims upon the Governor even for a few moments.

---

AT THE annual meeting of the regents of the University of the State of New York held at Albany, February 15, 1900, Mr. James Russell Parsons, Jr., was unanimously elected secretary, vice Melvil Dewey resigned. Mr. Parsons has been identified with the work of the regents office for a number of years. He was chiefly, the originator of the uniform system of licensing teachers, which is one of the excellencies of the New York State system of education.

In a paper read before the New York State Teachers' Association, at Elizabethtown, in 1887, he outlined a plan which was subsequently adopted. His presentation of the case was so clear that Andrew S. Draper, then State Superintendent of Public Instruction, decided to adopt it, and called Mr. Parsons into his office to organise the work. The success with which it was carried out is well known to every one familiar with education in this state.

The following appointments were also made by the regents at this meeting: Drs. William Warren Potter, of Buffalo; William S. Ely, of Rochester, and Maurice J. Lewi, of New York, nominated by the State Medical Society, and Asa S. Couch, of Fredonia; J. Willis Candee, of Syracuse, and John M. Lee, of Rochester, nominated by the Homeopathic Society, were appointed state medical examiners, to succeed themselves for a term of three years, from August 1, 1900.

The regents approved the plan of establishing at Alfred University a school of instruction in the sciences and technology of clay working and ceramics.

---

THE excellent meeting of the Medical Society of the State of New York recently held, was quite fully reported in the leading weekly medical journals. The *Journal* of the American Medical Association, however, was conspicuously silent in regard to it.

If the association journal desires to establish and maintain a reputation as a representative medical journal it can do so by exhibiting a spirit of fairness toward all reputable medical societies; especially by presenting reports of state society meetings that are of scientific value.

---

A BILL restraining the practice of midwifery in the City of New York by others than legally authorised physicians was introduced by Senator Plunkett, February 19, 1900. It provides that before July 1st the Board of Health of New York City shall appoint a board of examiners in midwifery. The board is required to examine all candidates twenty-one years of age and of good moral character who may apply for license to practise midwifery, and upon the receipt of $10, issue its certificate to those found qualified. Moneys thus collected are to be paid to the pension fund of the Department of Health of the city. Any person attempting to practise midwifery without such certificate shall be guilty of a misdemeanor, punishable by a fine of not less than $250 or more than $500.

---

FROM the report of the State Board of Medical Examiners representing the medical Society of the State of New York for the examination held in September, 1899, we glean the following statistics :

| | | |
|---|---|---|
| Total number of candidates | 162 | |
| " " " successful candidates | 127 | $78\frac{4}{10}$ per cent. |
| " " " unsuccessful " | 35 | $21\frac{6}{10}$ " " |

| | | | |
|---|---|---|---|
| Rejected in anatomy | 10 | Rejected in one topic only | 15—15 |
| " " physiology and hygiene | 15 | " " two topics | 7—14 |
| " " chemistry | 9 | " " three " | 4—12 |
| " " surgery | 12 | " " four " | 3—12 |
| " " obstetrics | 16 | " " five " | 1— 5 |
| " " pathology and diagnosis | 22 | " " six " | 1— 6 |
| " " therapeutics, practice and materia medica | 8 | " " seven " | 4—28 |
| | 92 | | 92 |

| | |
|---|---|
| Honor men | 8 |
| Highest general average | $93\frac{3}{10}$ |

---

BACTERIA in books is a contagion danger which cannot be too often referred to —one that circulating libraries subjects the public to. It is a common thing for books from such libraries to find their way into sick rooms, especially into those occupied by chronic invalids. The State Board of Health of Michigan reports the death from consumption of twenty department clerks whose work had been in certain volumes of records. The books were found by examining bacteriologists to be full of tubercle bacilli, and it is believed that they became infected from a clerk who had consumption, and who was known to have habitually moistened his thumb with saliva when he turned a page.

---

SURGEON-GENERAL STERNBERG has announced that acting assistant surgeons are needed in the Philippines. Applicants who are accepted must pass physical and professional examinations before contracts will be given them. In his announcement General Sternberg makes plain his position that only professional qualifications and endorsement will aid an applicant. Political pressure and letters will injure a candidate. Men between 25 and 35 are preferred, and they must have had experience in hospital or private practice.

THE medical students of Ohio are spending valuable time lobbying for a bill now before the legislature to do away with state examinations for a license to practise, in so far as it applies to graduates of medical colleges in Ohio.

Now, when in the course of the spring examinations, some of the students fall by the wayside, they will complain bitterly of the faculty. Their own waste of time and negligence will be the cause; not the severity of examination.

---

THE *Sanitary Bulletin* of New Hampshire shows in its January number that consumption in that state is on the decrease. In 1884, the death-rate was 14 per cent. It had decreased to 10 per cent. in 1899; increased during the influenza epidemic of 1890 to 11.5 per cent.; then fell to 9.5 in 1892; rose to a little over 10 per cent. in the three years following, falling last year to an even 9 per cent. No attempt is made to explain the cause of the decrease.

---

THE annual bulletin of the State Board of Health for 1899 shows that during the year just passed there were 121,320 deaths, an increase of 850 over 1898, and 4,740 more than 1897. The death-rate per thousand was 17.3, the same average which has been maintained for 10 years. Infant mortality and diphtheria show a decrease.

---

IOWA has come into line and all graduates of medical colleges will have to go before an examining board for a license to practise.

